# Epidemiological characteristics and prediction model construction of hand, foot and mouth disease in Quzhou City, China, 2005–2023

**DOI:** 10.3389/fpubh.2024.1474855

**Published:** 2024-12-18

**Authors:** Wenjie Xu, Canjie Zheng, Canya Fu, Xiaoying Gong, Quanjun Fang, Zhiying Yin

**Affiliations:** ^1^Department of Immunity, Quzhou Center for Disease Control and Prevention, Quzhou, Zhejiang, China; ^2^Department of Epidemiology, School of Public Health, Zhejiang Chinese Medical University, Hangzhou, Zhejiang, China

**Keywords:** HFMD, epidemiological, SARIMA, prophet, predictive model

## Abstract

**Background:**

HFMD is a common infectious disease that is prevalent worldwide. In many provinces in China, there have been outbreaks and epidemics of whooping cough, posing a threat to public health.

**Purpose:**

It is crucial to grasp the epidemiological characteristics of HFMD in Quzhou and establish a prediction model for HFMD to lay the foundation for early warning of HFMD.

**Method:**

Descriptive epidemiological methods were used to analyze the epidemic characteristics of HFMD, the incidence map was drawn by ArcGIS software, the Seasonal Auto Regressive Integrated Moving Average (SARIMA) and Prophet model were established by R software. Then, root mean square error (RMSE) and mean absolute error (MAE) were used to evaluate the fitting and prediction performances of the model.

**Results:**

From 2010 to 2023, Quzhou City reported a total of 66,601 cases of hand, foot, and mouth disease (HFMD), with the annual number of reported cases fluctuating between 2,265 and 7,964. The average annual incidence rate was 216.88/100,000, with the lowest in 2010 (97.08 /100,000) and the highest in 2016 (373.37/100,000) (*P*_trend_ < 0.001). The cases exhibited a seasonal bimodal distribution, with the first peak occurring from April to July and the second peak from October to December. The incidence rate of HFMD in males (246.71/100,000) was higher than that in females (185.81 /100,000). The performance of the SARIMA (1,0,1)(2,1,0)_12_ model was better than that of the Prophet model in terms of prediction accuracy.

**Conclusion:**

The incidence of hand, foot and mouth disease in Quzhou is on the rise in 2010–2016 and 2022–2023. In this study, the SARIMA prediction model was compared with the FB Prophet model. Data for more years can then be observed to better predict trends in the incidence of HFMD, providing a basis for prevention strategies and resource allocation. Further research can optimize the model to enhance predictive ability to improve the understanding and management of Chinese rival foot and mouth disease.

## 1 Introduction

HFMD (hand, foot and mouth disease) is a common infectious disease in children caused by enteroviruses. HFMD is prevalent worldwide (1), Since the 1990s, HFMD has resulted in thousands of deaths in the Asia-Pacific region (2). HFMD is prevalent in China (3), with outbreaks or epidemics reported in various provinces (4, 5). In May 2008, HFMD was included in the management reporting of Category C infectious diseases in China (6). Since 2010, HFMD has consistently ranked as the most prevalent statutory infectious disease in China. From 2008 to 2017, the average annual reported incidence of HFMD nationwide was 134.59 per 100,000 (7), making China one of the countries with a high burden of HFMD. Zhejiang Province has one of the highest incidences of Hand, Foot, and Mouth Disease in China. Studies indicate that from 2017 to 2023, the cumulative reported incidence of HFMD in Zhejiang ranked among the top five in the country (8). Quzhou City, located in the western part of Zhejiang Province, is a high-incidence area for Hand, Foot, and Mouth Disease. From 2016 to 2017, the incidence rate in Quzhou exceeded the provincial average for two consecutive years (9). In recent years, many researchers have employed time series analysis to study the temporal variations of HFMD and predict future trends, utilizing models such as the SARIMA (Seasonal Autoregressive Integrated Moving Average) model (10). This model has a simple structure and requires only incidence data for predictions, making it highly feasible. It has been widely applied in the field of healthcare (11), for example, pertussis (12), tuberculosis (13) prediction. However, a smooth time series is required, and differential operations can lead to excessive data stabilization. The Prophet model is a time series forecasting tool based on an additive model that can simultaneously capture multiple seasonalities through generalized additive models. It’s fitting performance has been validated in time series predictions for infectious diseases such as Hand, Foot, and Mouth Disease (HFMD), AIDS (14, 15), syphilis (16) and flu-like case (17) prediction. The advantage is that it is suitable for time series with seasonal and trend changes. Strong robustness with missing and outliers. The disadvantage is that the processing of non-stationary data is simple and may not be sufficient to handle complex non-stationary features. Many previous studies were based on a single model or method of disease prediction analysis. Prophet models validate fitting in time series predictions for infectious diseases such as HFMD (14, 15). SARIMA (12) is also widely used in infectious disease prediction. Such as hand, foot and mouth disease (18, 19), tuberculosis (20), new coronary pneumonia (11) etc. However, at present, there are few comparisons between SARIMA and prophet models for fitting and predicting the incidence of hand, foot and mouth disease. The prediction of HFMD by the two models carried out in this study complements the existing research and reinforces the research foundation.

Despite increased efforts in recent years to control the HFMD outbreak, the disease has been transmitted strong sexuality and complex transmission pathways still contribute to family and society heavy medical burden. To better understand the epidemiological characteristics of Hand, Foot, and Mouth Disease in Quzhou City, a descriptive analysis of HFMD data from 2005 to 2023 will be conducted. Utilizing GIS technology, the spatial characteristics of HFMD in Quzhou City over the years will be analyzed. Furthermore, SARIMA and Prophet models will be employed to establish predictive models for the reported incidence of HFMD from 2005 to 2023, comparing the predictive performance of each model.

## 2 Methods

### 2.1 Background

Quzhou is located in the western region of Zhejiang Province, China, situated upstream of the Qiantang River. It lies between longitudes 118°01′15″ and 119°20′20″ east and latitudes 28°15′26″ and 29°30′00″ north. The city’s topography is primarily mountainous and hilly, characterized by a subtropical monsoon climate, with an average annual temperature ranging from 16.3°C (in Kaihua) to 17.4°C (in the urban area). Quzhou experiences distinct seasons, ample sunshine, and abundant rainfall. The northern and southern mountainous areas receive more precipitation than the central plains, while the western region sees more rainfall than the eastern part. As of 2023, the city administers two districts, three counties, and one county-level city.

### 2.2 Data sources and collection

The HFMD case data for Quzhou City from January 2010 to December 2023 was obtained from the National Infectious Disease Reporting System (NIDRS) and the Chinese Center for Disease Control and Prevention (CDC). Cases were reported by various medical and health institutions at all levels in Quzhou and were reviewed by local disease prevention and control centers. All cases were confirmed according to the HFMD diagnostic criteria established by the Ministry of Health of the People’s Republic of China (WS588-2018).

### 2.3 Statistical analysis

#### 2.3.1 Descriptive analysis

Microsoft Excel (Microsoft Corporation, Redmond, W A, United States) was used to generate a database, organize and analyze the cases, and calculate the incidence rate. R software (version 4.2.1, R Foundation for Statistical Computing, Vienna, Austria) was used to analyze the prevalent characteristics of HFMD (time distribution, age distribution, sex distribution and occupation distribution) in Quzhou city from 2005 to 2023. Qualitative data were described as frequency and percent, and Chi-square test was used for inter-group comparison, with *p* < 0.05 indicating a statistically significant difference.

#### 2.3.2 Data processing

Get basic information on the incidence of hand, foot and mouth disease in Quzhou from NIDRS 2010–2023. After data cleansing, 66,601 case information was obtained. The incidence rate is defined as follows: Incidence = (number of new cases of a disease in a population in a given period/number of people exposed during the same period) × K, K = 100%, 1,000 ‰, 100 million or 100,000/100,000, etc. The number of onset per month according to the date of onset is included in the SARIMA and Prophet models analysis. Evaluate model fit performance with parameters such as MAE, MAPE and R^2^.The smaller the first two indicators the better, R^2^ the closer the better.


$$\begin{array}{l} MAE=\frac{1}{n}\overset{n}{\underset{i=1}{\sum }}|{\hat{y}}_{i}-y_{i}|\;\mathrm{MAPE}=\frac{100\%}{n}\overset{n}{\underset{i=1}{\sum }}|\frac{{\hat{y}}_{i}-y_{i}}{y_{i}}|\;\\ R^{2}=1-\frac{\sum_{i=1}^{n}\left(y_{i}-{\hat{y}}_{i}\right)2}{\sum_{i=1}^{n}\left(y_{i}-\bar{y}\right)2} \end{array}$$

#### 2.3.3 Model construction

The Autoregressive Integrated Moving Average (ARIMA) model, proposed by George Box and Gwilym Jenkins, is a classic time series forecasting method that has been widely applied in the field of public health (21–23). The ARIMA (p, d, q) model is a hybrid model composed of the Autoregressive (AR) component and the Moving Average (MA) component, where *p* represents the order of the autoregression, *d* is the order of differencing, and *q* denotes the order of the moving average.

The Seasonal ARIMA (SARIMA) model builds upon the ARIMA framework by incorporating seasonal parameters. In the SARIMA model, *P* is the order of the seasonal autoregression, *D* is the order of seasonal differencing, *Q* is the order of the seasonal moving average, and S represents the length of the seasonal cycle (24). The general form of the model is (p,d,q) × (P,D,Q)s, which is applicable to both trend and seasonal variations (25). This structure allows for the modeling of time series data that exhibit long-term trends as well as seasonal patterns.

The model construction process consists of four steps: (1) stationarity testing: the application of the SARIMA model requires that the time series meets the stationarity condition. Initially, the stationarity of the data is assessed through the time series plot. An augmented Dickey-Fuller (ADF) test is then conducted. If the *p*-value (*P*) is greater than 0.05 and the series is non-stationary, it is necessary to stabilize the original series through differencing and/or seasonal differencing, as well as variable transformations. (2) Model order identification: based on the characteristics of the autocorrelation function (ACF) and the partial autocorrelation function (PACF) plots, a preliminary determination of the order is made, and multiple models are compared. The optimal model is chosen according to the Akaike Information Criterion (AIC). (3) Parameter estimation and diagnosis: parameters of the identified model are estimated, and hypothesis testing is performed. The residual series undergoes a white noise test (Ljung-Box test). If the *p*-value *(P*) is greater than 0.05, this indicates that the model is suitable for forecasting. (4) Model fitting and forecasting: the final step involves fitting the model and making predictions. The SARIMA model selects the optimal fit model according to the comparison of information criterion methods. Select SARIMA by comparing different models, selecting the AIC, AICc, BIC minimum, *p* < 0.05, residual test passed as the optimal model (2,1,1)(0,1,1) (12). Make predictions for the optimal model.

The Prophet model is a time series forecasting tool developed by Facebook in 2017, implemented in the C++ programming language. It is available for use in both Python and R (26). The model can flexibly handle missing data and account for the effects of holidays and special events within the time series. It fits quickly, making it suitable for forecasting time series with strong seasonal influences and multiple seasonal historical data (14). Prophet Model Step Description: (1) The environment prepares for the installation of Prophet and the R package on which it depends. (2) Data preparation, with the number of cases of HFMD in order of month. Convert the dataset to the format required by Prophet, with the ds and y columns representing the date and prediction values respectively, split the dataset into training sets and test sets, and we use the last 12 months of data as test sets. (3) Model training, training Prophet models, creating a Prophet model object, fitting models with training set data, predicting future data using models. The parameters are set as follows:growth:liner, changepoint.range:0.9, yearly.seasonality:TRUE, seasonality.mode: multiplicative, interval.wideh:0.95. (4) Evaluating optimization, prophet provides a number of methods to evaluate the fit of a model, such as visualizing prediction results, calculating prediction errors, and so on. The general form of the Prophet model is given by y(t) = g(t) + s(t) + h(t) + *ε*, where g(t)represents the trend function, s(t)is the seasonal function, (h(t))accounts for holiday effects, and ε denotes the error term, which employs an additive model for accumulation. The model construction process mainly consists of four steps: model building, model evaluation, problem presentation, and visual feedback (15).

This study employs the selected optimal model to fit the HFMD case data from 2005 to 2022 and then forecasts the data for 2023. The model’s fitting and predictive performance are evaluated using the Root Mean Square Error (RMSE) and the Mean Absolute Error (MAE); smaller values of these metrics indicate better model performance. The SARIMA model is constructed using the R software (version 4.2.1, R Foundation for Statistical Computing, Vienna, Austria) with the “tseries” and “forecast” packages, while the “prophet” package is used to build the Prophet model.

## 3 Results

### 3.1 Descriptive statistics

**Figure 1:**
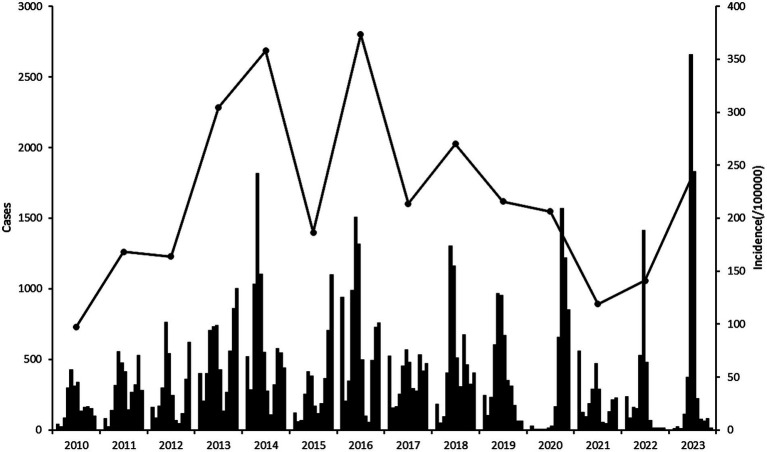
Cases and incidence rate of HMD in Quzhou City from 2010 to 2023.

From 2010 to 2023, Quzhou City reported a total of 66,601 HFMD cases, with the annual number of reported cases fluctuating between 2,265 and 7,964. The average annual incidence rate was 216.88/100,000 population, with the lowest rate recorded in 2010 (97.08/100,000) and the highest in 2016 (373.37/100,000). During the study period initially declining and then rising, with a statistically significant difference observed (*χ^2^* trend = 9234.9, *p* < 0.001) (Figure 1).

Among the HFMD cases, 38,650 were male (58.03%) and 27,951 were female (41.97%), resulting in a male-to-female ratio of 1.38:1. The incidence rate for males (246.71/100,000) was higher than that for females (185.81/100,000).

The age of onset ranged from 0 to 78 years, displaying a unimodal distribution, with the highest number of cases found in the 2-year age group, accounting for 32.66% (21,750 out of 66,601). The composition of cases varied across different age groups, with the highest incidence rate observed in the 1-year age group, followed by the 2-year age group, indicating a higher prevalence among younger children. The main occupations are Scattered Children 65.70% (43,760/66,601), Nursery Children 33.14% (22,069/66,601), School-age Children 1.11% (740/66,601) and Other 0.048% (32/66,601) (Table 1).

**Table 1:** Population distribution of HFMD in Quzhou City, 2010–2023.

Variables	Cases (*n* = 66,601)	Proportion (%)
Sex		
Male	38,650	58.03
Female	27,951	41.97
Age group (years)		
0-	5,916	8.88
1-	21,750	32.66
2-	15,764	23.67
3-	12,544	18.83
4-	6,184	9.29
5-	2,693	4.04
>5	1,750	2.63
Occupation		
Scattered children	43,760	65.70
Nursery children	22,069	33.14
School-age children	740	1.11
Other	32	0.05

### 3.2 Model prediction

**Figure 2:**
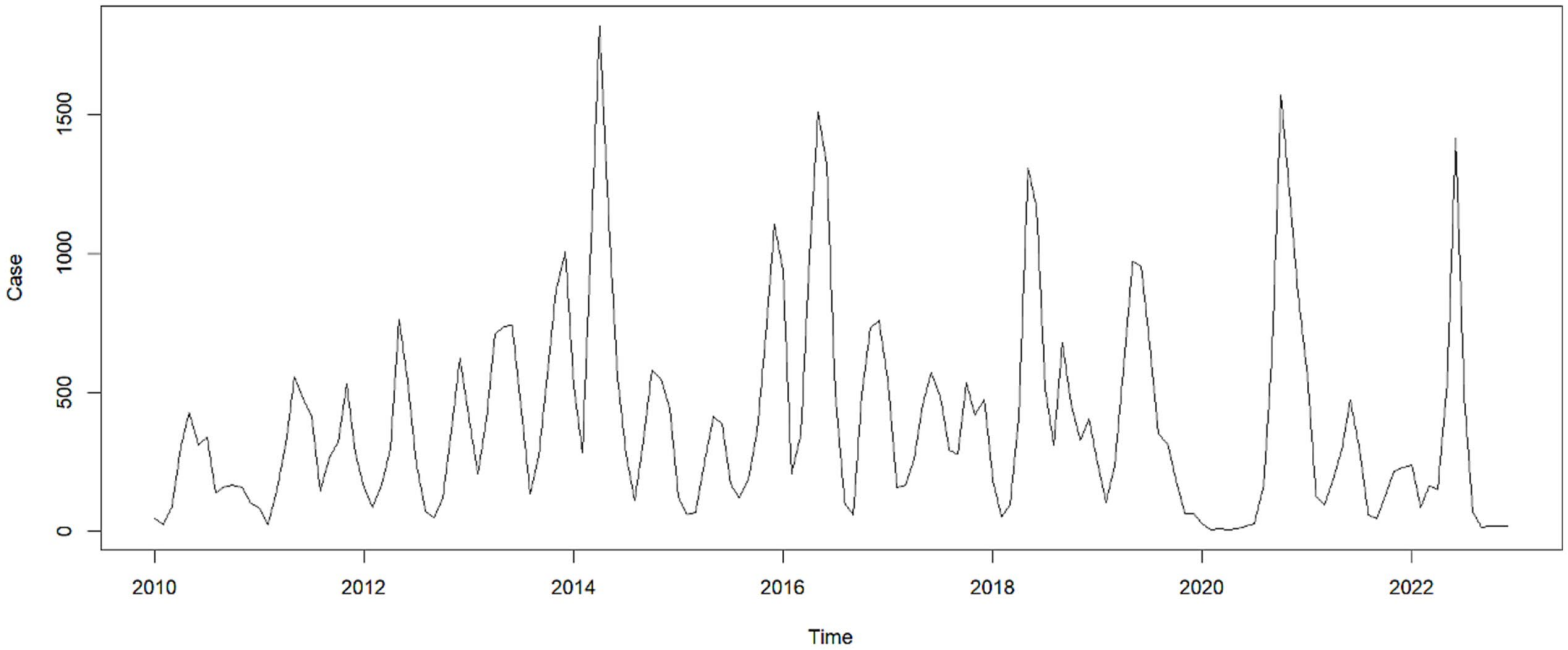
Time series of HFMD incidence in Quzhou City, 2010–2023.

**Figure 3:**
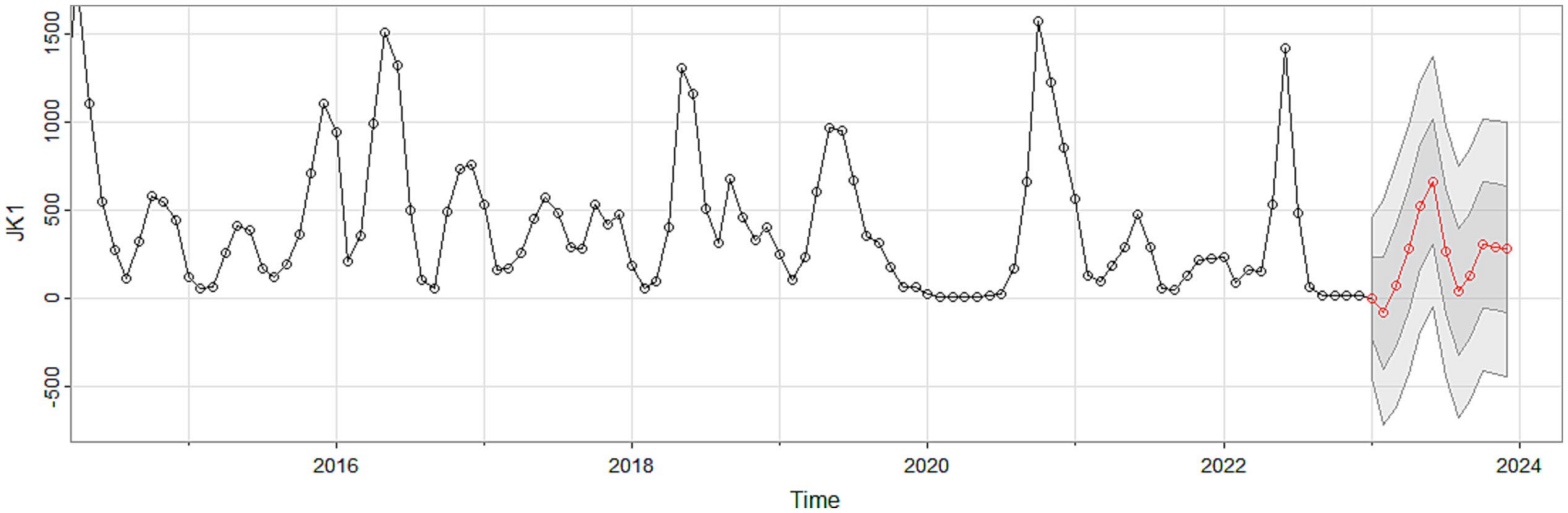
Time series decomposition of HFMD incidence in Quzhou City from 2010 to 2023.

**Figure 4:**
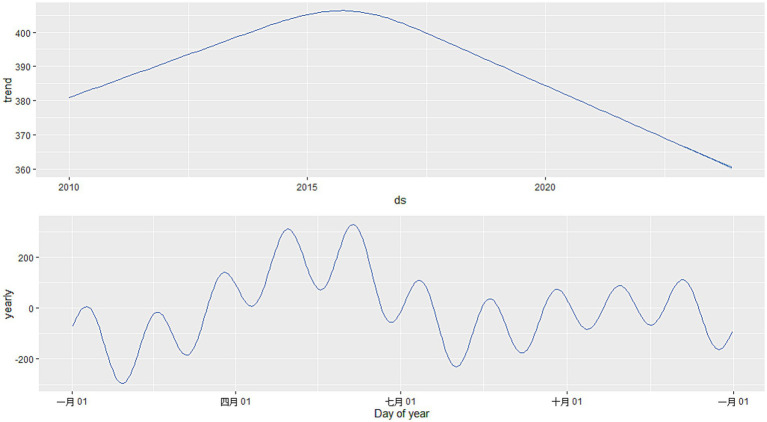
ACF after differencing transformation and PACF after differencing transformation.

**Figure 5:**
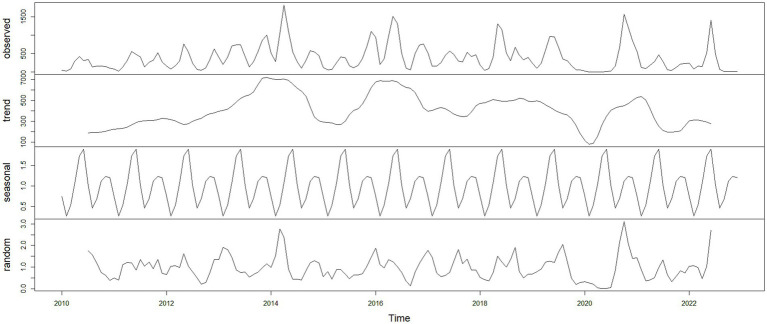
Residual diagnostics plots. Autocorrelation plot of the residual sequence; partial autocorrelation plot of the residual sequence; Ljung-Box test plot of the residual sequence.

**Figure 6:**
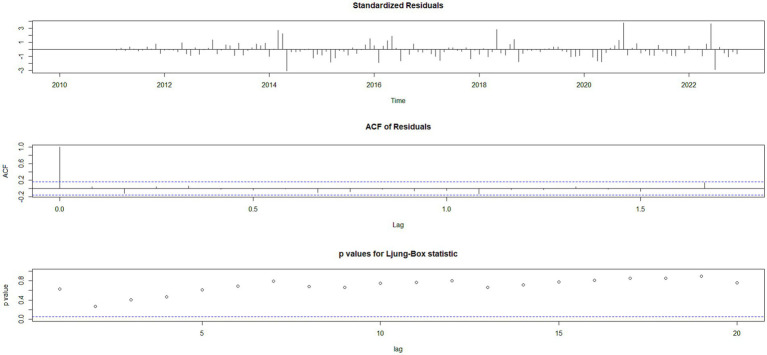
Fitting and forecast result of SARIMA.

Using the HFMD incidence data from Quzhou City from 2010 to 2022, a time series was established on a monthly basis. The time series plot (Figure 2) and its decomposition (Figure 3) indicate that the HFMD incidence rate in Quzhou City is stabilizing. The decomposition of the time series shows seasonal fluctuations, necessitating seasonal differencing of the original series. The ADF test (ADF = −5.8065, *p* < 0.05) confirms that the series is stationary, indicating parameters (*d* = 1) and (*D* = 1). The Box-Pierce test yielded a statistic of 5.6405 with a *p*-value <0.05.A seasonal model with a period of 12 months was simulated: SARIMA ((p, 1, q)(P, 1, Q)_12_). Autocorrelation and partial autocorrelation analyses (Figure 4) determined (*p* = 2) and (*q* = 1), with (P) and (Q) generally ranging from 0 to 2. By comparing models based on the information criterion, the model with the smallest AIC was selected, resulting in the optimal model being SARIMA ((2, 1, 1)(0, 1, 1) _12_). The results indicate that the model parameters are statistically significant (Table 2). Figure 5 shows that the ACF of the residuals lies within the upper and lower limits of two standard errors, and the *p*-values from the Ljung-Box test are significantly greater than the 0.05 significance level, suggesting that the residuals can be considered as a white noise series, indicating a good model fit. The SARIMA ((2, 1, 1)(0, 1, 1) _12_) model was used to fit the HFMD incidence trend in Quzhou City from 2010 to 2022. The overall trend of the actual values aligns with the fitted values, although the peak values did not fit well. The predicted HFMD case numbers for January to December 2023 were then compared to the actual monthly case numbers (Figure 6; Table 3).

**Table 2:** Parameter estimation and model verification of SARIMA model.

Parameters	Estimated value	Standard error	*t*-value	*p*-value
ar1	0.9270	0.0812	11.422191	<0.001
Ar2	−0.3425	0.0790	−4.336182	<0.001
ma1	−0.9508	0.0332	−28.641448	<0.001
sma2	−0.8099	0.0951	−8.520118	<0.001

**Table 3:** The monthly number of HFMD cases in the Quzhou City in 2023 predicted by the SARIMA, and Prophet models.

Month	Actual values	SARIMA model		Prophet model	
		Predicted values	95%CI	Predicted values	95%CI
1	11	2.70	(−225.09, 230.49)	285.03	(−127.16, 693.77)
2	25	−81.15	(−399.51, 237.21)	83.84	(−333.43, 444.09)
3	13	72.72	(−274.85, 420.29)	212.91	(−148.18, 596.15)
4	115	279.6	(−73.88, 633.08)	465.26	(59.85, 871.89)
5	375	525.10	(170.87, 879.33)	673.09	(265.82, 1052.44)
6	2,661	659.58	(305.21, 1013.95)	654.15	(230.04, 1041.54)
7	1,830	263.65	(−90.95, 618.25)	339.35	(−71.43, 756.1)
8	225	37.82	(−317.32, 392.96)	131.98	(−265.7, 524.58)
9	80	130.26	(−225.75, 486.27)	233.217	(−135.86, 615.94)
10	67	303.17	(−53.92,660.26)	400.53	(−1.03,795.98)
11	83	293.46	(−64.77, 651.69)	446.12	(28.13, 840.05)
12	18	279.98	(−79.37, 639.33)	456.33	(77.21, 865.43)

**Figure 7:**
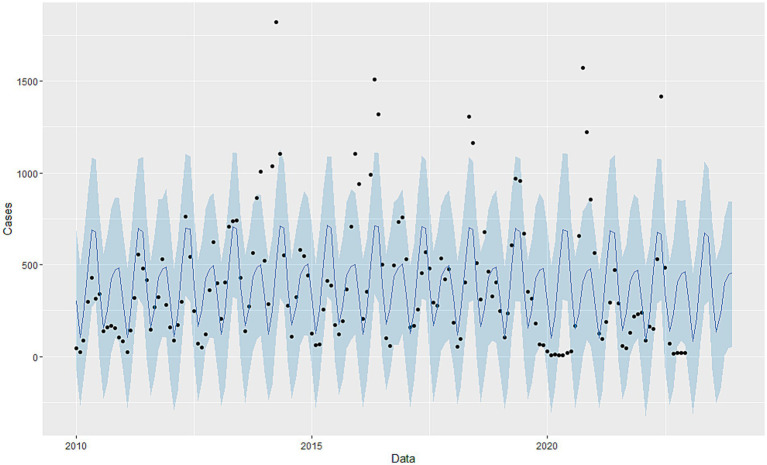
Decomposition of Prophet time series.

**Figure 8:**
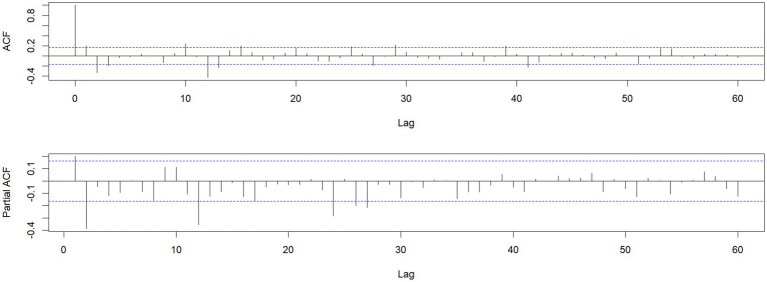
Fitting and forecast result of Prophet model.

Using the Prophet package in R, a Prophet model was constructed based on the monthly HFMD incidence data from Quzhou City from 2010 to 2022. The interval width was set to 0.95, while all other parameters were kept at their default values. The model incorporates “trend” and “year” time series components (Figure 7). Figure 8 illustrates that the model effectively captures the trend in HFMD incidence and achieves good results in forecasting HFMD data. Table 3 presents the predicted values and 95% confidence intervals for both the SARIMA and Prophet models. The results indicate that the RMSE and MAE values for the Prophet model on the test set are higher than those for the SARIMA ((2,1,1)(0,1,1)_12_) model (Table 4). This suggests that the performance of the SARIMA ((2,1,1)(0,1,1) _12_) model is superior to that of the Prophet model in terms of predictive accuracy.

**Table 4:** Comparison of the fitting and prediction performance of the two models.

Model	Validation Set		
	MAPE	MAE	*R* ^2^
SARIMA	0.75	8.30	0.61
Prophet model	24.91	274.03	0.43

## 4 Discussion

The results of this study indicate that from 2010 to 2023, the incidence of HFMD in Quzhou City showed a rising and then falling on the upward trend with a significant seasonal pattern. The peak incidence periods are observed from April to July and from October to December. The disease predominantly affects males compared to females, with the age group most affected being children aged 0–5 years. The distribution of cases is primarily among children in informal settings and daycare centers. Both SARIMA and Prophet models were employed for fitting and forecasting, with the SARIMA model demonstrating superior performance.

According to the Infectious Disease Prevention and Control Law of the People’s Republic of China, Hand, Foot, and Mouth Disease (HFMD) is classified as a Category C infectious disease in the country. Over the past few decades, its incidence has consistently ranked first among legally reportable infectious diseases (7). Before 2016, Hand, Foot, and Mouth Disease (HFMD) in Quzhou City was in a naturally occurring epidemic state, with annual reported incidence rates significantly higher than the national average. The incidence exhibited a “linear” upward trend prior to 2014, followed by a fluctuating decline after 2015. The peak incidence occurred in 2014, showing a similar trend to that of Guangxi. The annual reported incidence rate in Quzhou was lower than that in Guangxi (27), higher than that in Gansu (28), and comparable to that in Fujian (29).

After 2016, the reported incidence rate of Hand, Foot, and Mouth Disease (HFMD) exhibited a downward trend; however, it remained at a relatively high level. This could be attributed to changes in the dominant strains of the HFMD virus (30), as well as the introduction and application of HFMD vaccines in Quzhou City in 2016. Additionally, factors such as the local climate, the degree of social development, and population mobility in Quzhou may also play a role in influencing the incidence rates (31, 32).During the peak season for HFMD, some regions may also experience a minor autumnal peak in cases (33, 34). The reported incidence rate of HFMD in Quzhou City exhibits a seasonal bimodal trend, with a primary peak occurring from April to June and a secondary peak from October to December, similar to the patterns observed in Shaanxi Province (32).

This indicates that spring and autumn are critical periods for the prevention and control of Hand, Foot, and Mouth Disease (HFMD) outbreaks in Quzhou City. The variations in reported incidence rates across different seasons may be related to climatic factors affecting enteroviruses. Research has shown that the EV71 virus survives longer under conditions of low temperature and high humidity (35). Quzhou, being in a subtropical monsoon climate, experiences lower temperatures and more rainfall in spring and autumn, creating favorable conditions for the survival of enteroviruses. Analysis of the reported cases of Hand, Foot, and Mouth Disease (HFMD) in Quzhou City from 2010 to 2023 reveals that the incidence rate among children aged 0–5 years is notably high, accounting for 93.99% of all reported cases. This finding is consistent with research conducted in Gansu (28) and Jinhua (36).

The highest incidence rate is observed in children aged 1 year, which may be attributed to factors such as the natural decline of maternal antibodies after the age of one and increased exposure opportunities (37). In terms of gender, the number of cases in males exceeds that in females. Possible reasons for this include: first, boys are naturally more active and engage in outdoor activities more frequently than girls, which increases their chances of virus exposure and subsequent infection. Second, children under the age of five have weaker immune systems and are more susceptible to viruses. Additionally, children who are not yet in school lack education on hygiene practices, and caregivers may also have limited knowledge about Hand, Foot, and Mouth Disease, resulting in a lack of awareness and preventive measures, thereby increasing the risk of infection (38). So we give the following clinical recommendations: Firstly, key areas and key population monitoring programmes are developed to ensure timely detection and management of outbreaks. Secondly, we need to optimize the allocation of medical resources, improve the level of medical institution construction, improve the diagnosis and treatment ability of hand, foot and mouth disease. Furthermore, the EV71 vaccine is effective in preventing hand, foot and mouth disease caused by EV71 infections. Therefore, we should further promote the vaccination of children of appropriate age EV71 and improve the immunity of the population. In addition, continuous improvement of environmental hygiene, with particular attention to hygiene management in child-intensive places such as schools and kindergartens, and regular preventive disinfection to reduce the chances of transmission of the virus. At the same time, to raise public health awareness through media publicity, health education activities and other ways to popularize the knowledge of hand, foot and mouth disease prevention and control, encourage parents to strengthen daily observation, once the discovery of suspected symptoms, immediately seek medical treatment.

This study is based on the HFMD incidence data from Quzhou City from 2010 to 2022, in which we constructed SARIMA and Prophet models to fit and predict the trends of HFMD incidence. We found that the optimal ARIMA model was more effective, with lower MAE, MAPE and higher *R*^2^ evaluation metrics compared to the Prophet model, indicating smaller prediction errors. Perhaps the model we studied was predictive of instability, and the reason for the analysis was probably the effect of: Firstly, meteorological factors. The ARIMA model only considers the effects of temporal factors on morbidity. Studies have shown that the incidence of HFMD is associated with average daily temperature, average daily humidity, and precipitation (39). Later studies may attempt to add meteorological factors to further correct the model. Secondly, The ARIMA model is a linear model in principle. But the incidence of infectious diseases is generally non-linear. This causes some limitations in model fitting. Resulting in errors, the study only divided the data into training and prediction samples. May cause overfitting or underfitting (40). Thirdly, Social factors. Infants and young children are susceptible to hand-foot-and-mouth disease. When the second-child policy is liberalized, the birth rate rises over a short period of time and decreases gradually (41). Our study also has limitations. First, the HFMD case data were collected through passive surveillance, which may introduce biases such as underreporting. Second, the model construction only considered the intrinsic characteristics of the time series and did not account for other exogenous variables, such as meteorological factors and economic variables. Thirdly, since the research data are specific to Quzhou City in China, our conclusions are only applicable to the population in that region, limiting the generalizability of the results. In the future, we need to further explore the interactions within the SARIMA model, considering various influencing factors to achieve more realistic predictions and using the ensemble technique combining multiple prediction models into a single ensemble model to improve the stability and accuracy of predictions. The reason behind the differences in model performance may be that we do not take into account the factors that affect the occurrence of HFMD, such as pathogens, hosts, natural environments, vaccines and socioeconomics. The disease prediction model of this study did not take into account the effect of the changes in the cases of new coronary infection against foot and mouth disease. It may not be possible to provide a basis for more comprehensive health policy formulation.

## 5 Conclusion

From 2010 to 2023, the overall incidence of Hand, Foot, and Mouth Disease (HFMD) in Quzhou City showed a declining trend; however, the epidemic remains severe in Kecheng District and Kaihua County. Infants and young children should be the primary focus of HFMD prevention and control efforts. In the future, it is important to continue enhancing the monitoring of HFMD dynamics, conduct health education initiatives before the high incidence season, and promote the vaccination of eligible children with the EV71 vaccine. The SARIMA model effectively fits and predicts the incidence trends of HFMD in Quzhou City, providing early warnings and forecasts for HFMD occurrences and offering reliable data for the prevention and control of HFMD in the region.

The overall incidence of HFMD in Quzhou City showed a trend of decline followed by an increase from 2010 to 2023, with a particularly severe situation in Kaihua County. Male children should be a key focus group for HFMD prevention and control. In the future, it is essential to continue strengthening the dynamic monitoring of HFMD outbreaks. Scientific prevention and control measures should be implemented before the peak period of HFMD, promoting vaccination and health education, especially for younger children. The SARIMA model has effectively fitted and predicted the occurrence trend of HFMD in Quzhou, providing forecasting for HFMD cases in the city.

## Data Availability

The original contributions presented in the study are included in the article/supplementary material, further inquiries can be directed to the corresponding author.
